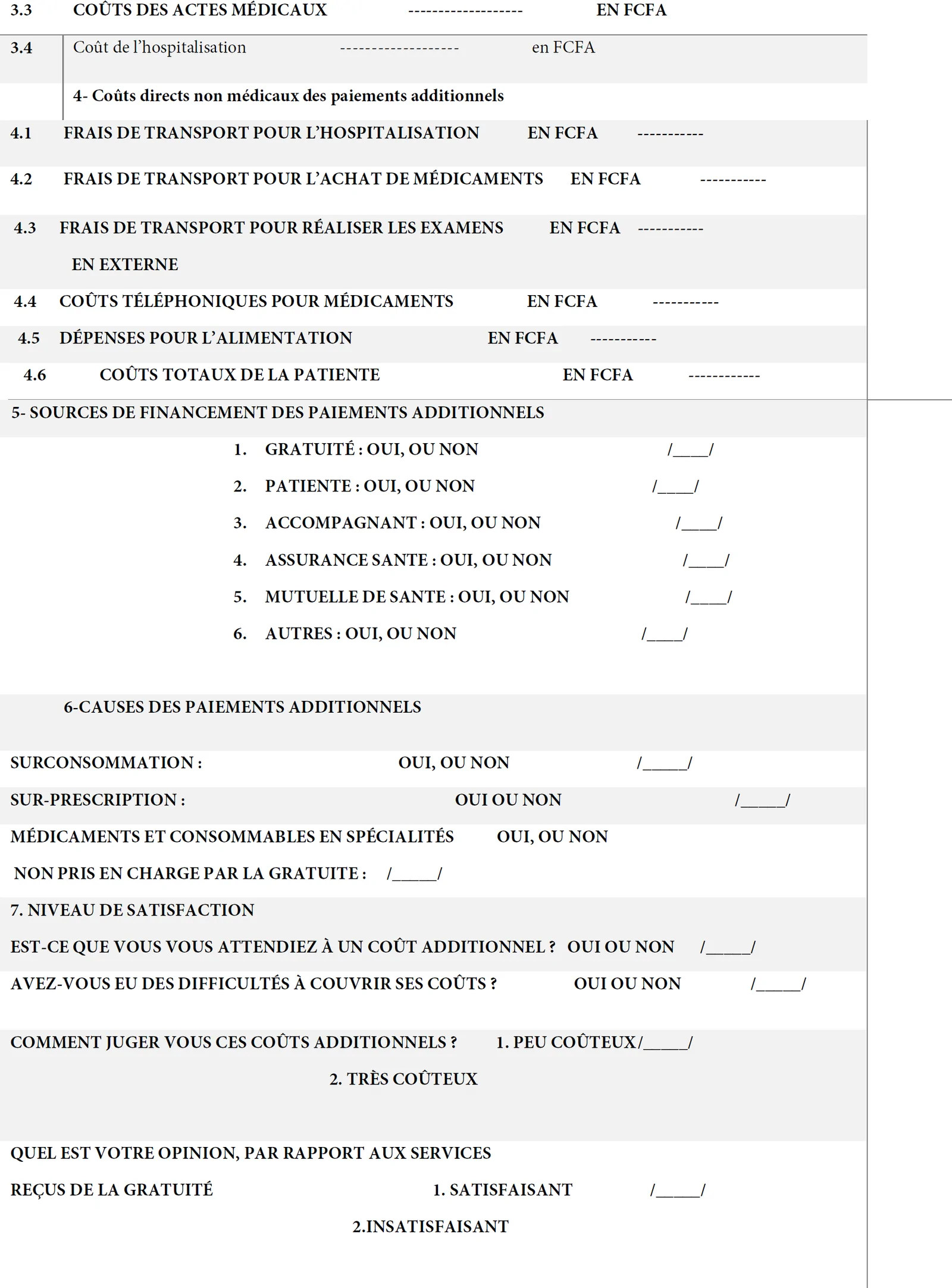# Coûts additionnels payés par les femmes enceintes bénéficiaires de la gratuité des soins au CHU Sourô SANOU de Bobo-Dioulasso (Burkina-Faso)

**DOI:** 10.48327/mtsi.v6i3.2026.686

**Published:** 2026-09-28

**Authors:** Seydou TRAORÉ, Laure Miriam Boudnoma YAMÉOGO, Ziemlé Clément MÉDA

**Affiliations:** 1 Département de santé publique, Université NAZI BONI, Bobo-Dioulasso, Burkina Faso; 2 Pharmacienne généraliste indépendante

**Keywords:** Gratuité des soins, Femmes enceintes, Accouchées récentes, Coûts additionnels, Bobo-Dioulasso, Burkina Faso, Afrique subsaharienne, Free healthcare, Pregnant women, Postpartum, Additional costs, Bobo-Dioulasso, Burkina Faso, Sub-Saharan Africa

## Abstract

**Introduction:**

Le Burkina-Faso a mis en œuvre en 2016 la gratuité des soins dans les structures sanitaires publiques chez les femmes enceintes et les accouchées récentes. Cependant, certaines dépenses ne sont ni couvertes ni remboursées. L’objectif de cette étude est d’estimer ce coût additionnel.

**Méthode:**

Il s’est agi d’une étude transversale à visée descriptive et prospective des dépenses de santé additionnelles payées par les femmes enceintes bénéficiaires de la gratuité des soins. Les patientes ont été incluses de façon consécutive. Les données ont été recueillies pendant un mois.

**Résultats:**

L’étude a concerné 220 femmes enceintes et accouchées récentes parmi lesquelles 180 (soit 81,8 %) ont dû s’acquitter d’un paiement additionnel. La moyenne d’âge de ces dernières était de 27 ans (IC 95 % 25-31). Le motif d’admission le plus fréquent était l’accouchement (66,7 %) dont la majorité était eutocique (57,5 %). Le coût direct médical moyen était de 7 693 FCFA (IC 95 %, 6 565-8 821) et représentait 7,2 % du coût direct total. Le coût direct non médical moyen était de 5 127 FCFA (IC 95 % 4 738-5 516). Le coût additionnel total moyen était de 10 356 FCFA (IC 95 %, 9 186-11 527). Le coût direct total additionnel représentait 13,4 % du coût direct total. La majorité (70 %) des femmes n’était pas préparée à des coûts additionnels en se rendant à l’hôpital et 65,6 % d’entre elles ont eu des difficultés pour les honorer. Plus de la moitié (55,6 %) était insatisfaite du non-respect de la gratuité des soins.

**Conclusion:**

Les coûts additionnels subsistent malgré l’instauration de la gratuité des soins et compromettent la prise en charge des femmes les plus pauvres.

## Abstract (en)

**Introduction:**

In 2016, Burkina Faso began offering free healthcare at public health facilities to pregnant women and new mothers. However, certain expenses are neither covered nor reimbursed. This study aims to estimate these additional costs.

**Methods:**

This cross-sectional, descriptive, prospective study examined the additional healthcare expenses paid by pregnant women and were beneficiaries of the free healthcare program. Patients were enrolled consecutively. Data were collected over a one-month period.

**Results:**

The study included 220 pregnant and postpartum women, 180 of whom (81.8%) had to make an additional payment. The mean age of these women was 27 years (95% CI, 25–31 years). The most common reason for admission was childbirth (66.7%), most of which were uncomplicated deliveries (57.5%). The average direct medical cost was 7,693 CFA francs (95% CI: 6,565–8,821), accounting for 7.2% of the total direct costs. The average direct non-medical cost was 5,127 CFA francs (95% CI: 4,738–5,516). The average total additional cost was 10,356 CFA francs (95% CI: 9,186–11,527). The total additional direct costs accounted for 13.4% of the total direct costs. Most women (70%) were not prepared for the additional costs associated with going to the hospital, and 65.6% had difficulty paying them. More than half (55.6%) were dissatisfied with the lack of free care.

**Conclusion:**

Despite the introduction of free healthcare, additional costs persist and compromise the care of the poorest women.

**Figure:**
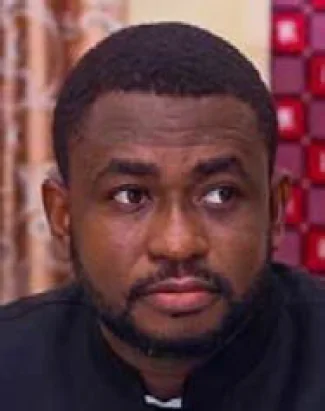

## Introduction

En 1993, le Burkina Faso a généralisé le système de paiement direct issu de l’initiative de Bamako dont l’objectif principal était la recherche de l’autonomie financière des districts. Les frais de consultations et les médicaments essentiels vendus dans les pharmacies des centres de santé devenaient payants. Ces sommes servaient à l’achat des médicaments et devaient en principe améliorer la qualité des soins [17].

Depuis plusieurs années, le système de santé du pays a montré ses limites avec une couverture sanitaire parmi les plus faibles au monde.

Parmi les pays de l’Afrique de l’Ouest qui ont adopté l’initiative de Bamako, rares sont ceux qui avaient plus de 0,5 consultation par an et par habitant [14]. Il était impératif de développer des initiatives pour améliorer l’accès aux soins dans ces pays.

C’est dans cette logique que s’inscrit la politique de gratuité des soins. Au cours des années 2000, les politiques de subvention et d’exemption se sont multipliées mais souvent sans s’intégrer dans un programme coordonné avec une vision à long terme. Les programmes de gratuité des soins ont été aussi parfois mis en œuvre pour l’obtention des subventions auprès des institutions internationales [15].

Les bailleurs de fonds ont fait de l’amélioration de la santé maternelle et infantile une priorité majeure de la communauté internationale devenue une cible à part entière des objectifs du développement durable (ODD 2023) [6].

Le Burkina Faso s’est engagé à atteindre ces objectifs. Cela passe par la mise en œuvre de réformes majeures dans le secteur de la santé. Dans un pays où 45 % de la population vit en dessous du seuil de pauvreté et en l’absence d’un régime d’assurance maladie, il était nécessaire de développer des politiques adaptées pour permettre aux populations concernées d’avoir accès aux soins [8].

C’est ainsi que le gouvernement a promu la gratuité des soins pour les femmes enceintes et les enfants de moins de cinq ans à partir de 2016 [14]. Cette gratuité prévoit la prise en charge des actes de soins, d’hospitalisation, des examens et des médicaments. Elle comprend le dépistage et le traitement des lésions précancéreuses du col de l’utérus, tels que l’inspection visuelle après application d’acide acétique (IVA) ou de solution de Lugol (IVL), la cryothérapie et la résection par anse diathermique. Elle inclut également le coût des services de planning familial, y compris la prise en charge des effets secondaires liés à la planification familiale (PF), ainsi que les soins pendant la grossesse, l’accouchement et les interventions obstétricales. Lors des soins curatifs, la femme enceinte bénéficie de tous les examens nécessaires à sa prise en charge et disponibles dans les centres de santé mettant en œuvre la gratuité, y compris les frais liés à l’évacuation vers le centre de santé [14].

La gratuité des soins a pour objectif d’améliorer la fréquentation des centres de santé par les bénéficiaires [7,16,18]. Malgré cette gratuité, certaines bénéficiaires continuent de payer des frais pour des soins normalement pris en charge. Cette situation survient lorsque les médicaments prescrits ou les examens demandés aux patientes ne sont pas disponibles dans la structure de santé concernée. Cela les oblige à payer ces médicaments dans les officines privées ou à réaliser les examens concernés dans les cliniques ou laboratoires privés [21]. L’objectif de cette étude était d’évaluer l’importance du coût additionnel payé par les femmes enceintes bénéficiaires de la gratuité théorique des soins dans un établissement public spécialisé de niveau 3.

## Matériels et méthodes

Il s’est agi d’une étude monocentrique descriptive et à collecte prospective des dépenses de santé additionnelles payées par les patientes bénéficiaires de la gratuité des soins au Centre hospitalier universitaire Sourô Sanou (CHUSS) du Burkina Faso. Ce CHU est un établissement public spécialisé de niveau 3, retenu en raison de la qualité de l’offre de soins curatifs qu’il propose dans la pyramide sanitaire du pays. Il s’agissait d’évaluer le coût des dépenses du point de vue des patientes.

La taille d’échantillon a été déterminée à partir de la formule classique d’estimation d’une proportion en population. En l’absence d’information préalable sur la prévalence recherchée, on a retenu la situation la plus défavorable à 50 %, le niveau de confiance à 95 % et la marge d’erreur à 7 %. Ce dernier choix a été fait dans le contexte d’une étude observationnelle menée en milieu hospitalier, caractérisée par une population source limitée et des contraintes logistiques de collecte, soit environ 196 femmes à inclure. Nous avons choisi un taux de non-réponse de 10 % et avons estimé à 220 le nombre total de patientes.

Nous avons inclus uniquement les femmes bénéficiaires de la gratuité des soins de manière consécutive durant la période de collecte. Il s’agis-sait des femmes enceintes ou accouchées récentes (moins de 42 jours), âgées de plus de 18 ans, admises dans le service d’obstétrique du département de gynécologie, d’obstétrique et de médecine de la reproduction (DGOMR) du CHUSS. Elles devaient avoir donné leur consentement pour participer à l’étude et disposer de fiches de collecte complètes. Les patientes qui ont été transférées dans un autre service pour la suite de la prise en charge ont été exclues de l’étude.

La collecte des données a été faite de manière prospective durant une période d’un mois, du 1er au 31 octobre 2024. Les patientes ont été incluses durant leur séjour à l’hôpital. La fiche de collecte a été renseignée quotidiennement depuis l’admission jusqu’à la sortie pour ce qui concerne les prescriptions faites au quotidien. Le total a été fait à la sortie.

Le flux des participantes à l’étude est présenté à la Figure 1.

**Figure 1:**
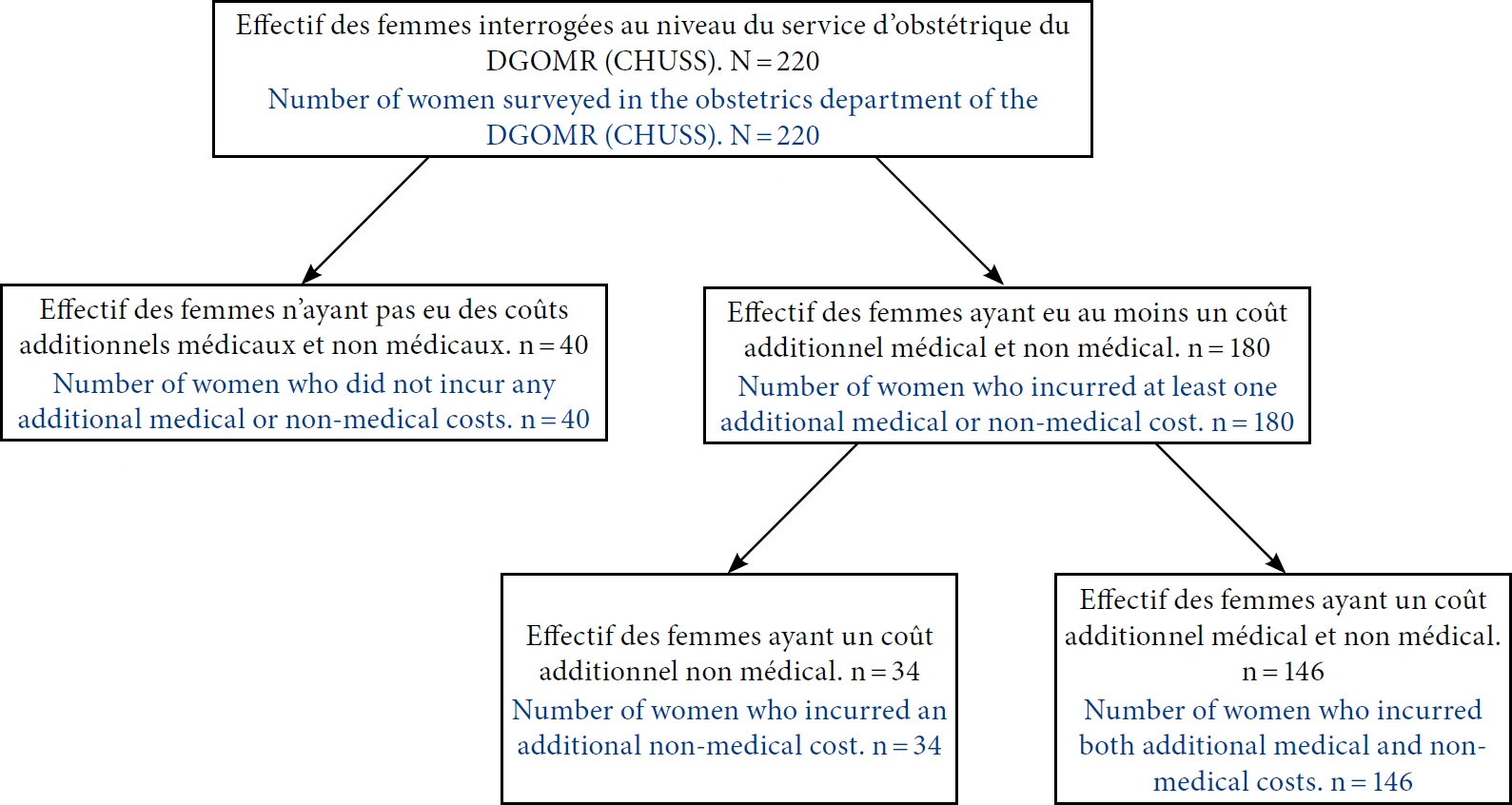
Flux des femmes bénéficiaires interrogées et celles incluses dans l’étude

Les données ont été collectées à l’aide d’une fiche (Annexe 1). Le questionnaire a été administré sous forme de support papier rempli par nousmêmes au cours d’un entretien individuel auprès des femmes bénéficiaires de la gratuité et répondant à nos critères d’inclusion.

Les sources retenues étaient : l’interrogatoire des patientes, les registres de consultation, les dossiers cliniques des patientes hospitalisées, les registres d’hospitalisation, les reçus et quittances de paiement et les déclarations des parents. Pour les dépenses de santé, nous avons consulté les reçus de caisse pour les médicaments et les examens payés hors de la gratuité des soins.

Les coûts moyens ont été calculés en rapportant la somme des coûts pour une patiente à l’effectif interrogé concerné.

Les frais de transport ont été estimés au prix du kilomètre rapporté au nombre de kilomètres parcourus par le patient.

Les différents coûts ont été définis de la manière suivante :

- Coût direct total = coûts directs médicaux + coûts directs non médicaux;
- Coût direct médical = coût du diagnostic + coût du traitement;
- Coût du diagnostic = coût des consultations diagnostic + coût des examens diagnostic;
- Coût du traitement = coût des médicaments et dispositifs médicaux ayant servi à la prise en charge;
- Coût direct non médical = coût du trans-port pour aller chercher les médicaments et consommables inclus normalement dans la gratuité et qui ne sont pas disponibles à l’hôpital + coût de l’alimentation + coût de l’hébergement + coût du crédit téléphonique (pour contacter les pharmacies de villes et les laboratoires privés pour la disponibilité des médicaments et des examens normalement pris en charge) + coût des autres dépenses annexes aux soins;
- Coût additionnel des soins de santé : l’en-semble des coûts directs totaux qui devraient être pris en charge par la gratuité mais qui, pour diverses raisons, sont assumés par les patientes elles-mêmes. Ces frais concernent les coûts directs médicaux et les coûts directs non médicaux.

L’analyse a été faite à l’aide du logiciel R studio et du logiciel Excel dans sa version 2019.

Avant d’entreprendre cette étude, nous avons obtenu l’accord du comité de thèse de l’université Nazi Boni et l’autorisation N° 2024-431/MSHP/ SG/CHUSS/DRH de la direction du CHUSS. La participation à l’étude, librement acceptée et exprimée oralement par chaque patiente, ne donnait droit à aucun avantage et le refus de participer n’avait aucun impact sur la continuité des soins.

## Résultats

L’échantillon étudié était constitué de 220 femmes. Parmi elles, 180 ont eu à payer des coûts additionnels. Pour 34 d’entre elles, il s’agissait uniquement de coût additionnel non médical et pour les 146 autres, de coût additionnel médical et non médical. L’âge moyen était de 27 ans (IC 95 % 25-31) avec des extrêmes de 18 à 43 ans. Les femmes résidant en milieu urbain représentaient 84,4 % de l’effectif total des 180 femmes étudiées. Celles qui avaient tout au plus un niveau d’instruction primaire représentaient 80,6 %. La majorité des femmes (51,7 %) étaient des femmes au foyer, mariées dans leur majorité (82,8 %) et dont le motif d’hospitalisation était principalement l’accouchement (67 %). Les diagnostics les plus fréquents étaient respectivement les accouchements eutociques (38,3 %) et les disproportions fœtopelviennes (28,3 %). Le Tableau I donne la répartition des patientes par diagnostic principal. Les femmes venues d’ellesmêmes représentait 91,1 % de l’effectif. La durée moyenne d’hospitalisation était 2,9 jours (IC 95 % = 2-3,6) avec des extrêmes allant de 1 à 13 jours. La majorité des femmes sont sortie avec un avis médical favorable (97,8 %).

**Tableau I:** Répartition des diagnostics posés

Diagnostics	Effectifs (n)	%
Disproportion fœto-pelvienne	51	28,3
Eutocie	69	38,3
Hémorragies	3	1,7
Infections*	35	19,4
Prééclampsie/éclampsie	10	5,6
Autres**	12	6,7
**Total**	**180**	**100,0**

* paludisme, dengue, fièvre typhoïde

* anémie, avortement spontané, crise drépanocytaire, diabète, grossesse extra-utérine, grossesse molaire, hypotension et thrombose veineuse

Le nombre total d’ordonnances prescrites était de 1 057, dont 79,6 % ont été honorées totalement et 20,4 % partiellement par les patientes. Au total, 1874 médicaments, kits ou consommables médicaux ont été prescrits aux 180 patientes avec une moyenne de 11,4 par personne. Sur les 896 médicaments prescrits, plus des ¾ étaient des médicaments génériques (76,1 %).

Le nombre moyen de médicaments payés hors de la gratuité était de 2,2 (IC 95 % 1,64-2,84) pour un nombre minimum de 1 et un nombre maximum de 25.

On dénombrait 267 médicaments et consommables médicaux payés par les patientes, soit 14,2 % de l’ensemble des prescriptions.

Le coût direct médical additionnel moyen était de 7 693,3 FCFA^1^ (IC 95 % = 6 565-8 821), le coût médian de 5800 FCFA (IIQ : 3 170-10 100). Les coûts additionnels représentaient 12,5 % du coût direct total dépensé pour les médicaments et consommables, et 2,5 % du coût total des examens. Aucun coût additionnel sur les actes médicaux n’est apparu. Le coût moyen des examens était de 7 477,4 FCFA (IC 95 % = 6 359-8 596).

Seulement 4 examens ont été réalisés hors de la gratuité pour un coût moyen de 8 400 FCFA (IC 95 % = 4 645-12 154).

Les coûts additionnels représentaient 7,2 % des coûts directs médicaux totaux.

Le Tableau II donne la répartition des coûts directs médicaux additionnels.

**Tableau II:** Récapitulatif des coûts directs médicaux additionnels en FCFA

Type de coûts directs médicaux additionnels	Effectifs (n)	Moyenne	Écart type	IC 95 %
Coûts totaux des médicaments et consommables médicaux honorés hors gratuité	120	7 477,4	6 250,3	[6 359- 8 595]
Coûts directs médicaux totaux additionnels	121	7 693,3	6 331,4	[6 565- 8 821]

Les frais de transport pour hospitalisation, la réalisation des examens complémentaires et l’achat des médicaments représentaient 34 % des coûts directs non médicaux totaux. Le coût moyen du transport pour l’hospitalisation était de 1 247 FCFA (IC 95 % = 1 125-1 368). Seulement quatre patientes ont déclaré avoir payé le transport pour l’achat des médicaments. Le coût moyen des dépenses de transport pour achat des médicaments était de 879 (IC 95 % = 792-966). Le Tableau III précise la répartition des coûts directs non médicaux additionnels.

**Tableau III:** Récapitulatif des coûts directs non médicaux additionnels en FCFA

Type de coûts directs non médicaux additionnels	Effectifs (n)	Moyenne	Écart type	IC 95 %
Frais de transport pour hospitalisation en FCFA	164	1 246,9	793,9	[1 125-1 368]
Frais de transport pour achat des médicaments en FCFA	120	879	486,1	[791-966]
Coûts téléphoniques en FCFA	84	704	301,8	[639-769]

Dans leur grande majorité, les patientes ont eu leurs coûts additionnels financés par les accompagnateurs (94,4 %). Dans la plupart des cas (70 %), elles n’étaient pas préparées à un coût additionnel, et plus de la moitié a eu des difficultés financières à honorer ces coûts (65,6 %). Une grande partie des femmes bénéficiaires de la gratuité déclarait que les coûts additionnels étaient très élevés (66,7 %) et 55,6 % se déclaraient insatisfaites des services reçus. Les femmes insatisfaites ont eu les coûts directs médicaux et non médicaux des paiements additionnels les plus élevés. Le Tableau IV récapitule cette relation.

**Tableau IV:** Relation entre les coûts directs médicaux et non médicaux additionnels en FCFA et opinion des femmes sur les services rendus

Opinion des bénéficiaires	Effectifs (n)	Moyenne des coûts directs médicaux et non médicaux additionnels	IC95 %	p
Insatisfaite	100	15 418,5	[10 824,1 - 20 012,9]	< 0,001
Satisfaisante	80	3 900,4	[2 145,7 - 5 655,1]	
Total	180	10 299,4	[7 682,3 - 12 916,5]	

## Discussion

Cette étude visait à estimer le coût additionnel supporté par les femmes enceintes bénéficiaires de la gratuité des soins au CHU Sourô SANOU de Bobo-Dioulasso.

La gratuité des soins au Burkina Faso couvre en principe le dépistage et le traitement des lésions précancéreuses du col utérin, la planification familiale, le suivi de grossesse, l’accouchement et les interventions obstétricales, ainsi que les examens complémentaires nécessaires disponibles dans les centres de santé et les frais d’évacuation [14]. Les examens réalisés hors de l’hôpital et les médicaments achetés en pharmacie privée par les bénéficiaires devraient donc, en théorie, être intégralement pris en charge, ce qui n’a pas été le cas dans notre série.

Cet écart s’explique en partie par le contexte de mise en œuvre du programme. Décidée dans un contexte politique marqué par l’instabilité de 2014-2015 et présentée comme une promesse électorale tenue, la gratuité des soins a été une décision d’abord politique avant d’être technique, instaurée sans réelle concertation préalable avec les acteurs de terrain à savoir les soignants, gestionnaires et bénéficiaires. Son extension progressive aux formations sanitaires privées et confessionnelles conventionnées repose sur un mécanisme de remboursement par l’État, aujourd’hui marqué par des retards qui fragilisent le réapprovisionnement des structures. La Centrale d’achat des médicaments essentiels génériques pallie en partie cette difficulté en fournissant médicaments et dispositifs médicaux à crédit, mais les ruptures qu’elle connaît elle-même, ainsi que les pannes récurrentes des équipements de laboratoire et d’imagerie, contraignent régulièrement les patientes à se procurer médicaments et examens dans le secteur privé.

Le recueil prospectif des dépenses, avec vérification des pièces justificatives chaque fois que possible, constitue un atout méthodologique: il a permis de limiter le biais de mémorisation inhérent aux enquêtes rétrospectives sur les dépenses de santé.

L’étude comporte cependant des limites. D’abord, elle repose uniquement sur les informations rapportées par les patientes, sans triangulation systématique avec la comptabilité des formations sanitaires, ce qui expose à un biais de déclaration. Ensuite, l’absence de comptabilité exhaustive des dépenses et la diversité des accompagnateurs chargés des achats ont pu conduire à une sousestimation des coûts directs; ce biais a été atténué, sans être totalement éliminé, par la vérification des justificatifs disponibles. Enfin, l’étude a été menée dans un seul des cinq hôpitaux universitaires du pays, ce qui limite l’extrapolation des résultats à l’ensemble du territoire, même si des pratiques comparables ont été rapportées dans d’autres structures. Malgré ces limites, nous avons pu estimer les coûts résiduels payés par les bénéficiaires de la gratuité des soins.

Nos résultats montrent que, malgré cette politique, la grande majorité des femmes a dû engager des dépenses supplémentaires, non médicales comme médicales, d’un montant non négligeable au regard des ressources des ménages concernés. Cette persistance de paiements directs, plus fréquente et plus coûteuse chez les femmes les moins préparées financièrement, confirme que la gratuité, telle qu’elle est actuellement mise en œuvre, ne couvre pas l’ensemble des besoins des bénéficiaires et compromet en particulier l’accès aux soins des femmes les plus pauvres.

L’existence de paiements directs malgré des politiques de gratuité a été rapportée dans plusieurs pays d’Afrique subsaharienne, avec des amplitudes variables selon le contexte. Au Ghana, Dalinjong *et al*. ont relié ce phénomène au manque d’intrants et d’infrastructures, traduisant une allocation de ressources insuffisante aux établissements de soins [5]. Au Burkina Faso, l’augmentation croissante des dépenses de la gratuité des soins depuis sa mise en œuvre va se traduire par une insuffisance des dotations budgétaires du gouvernement [20]. En effet, en 2024, le taux de couverture financière des prestations de la gratuité des soins par l’état était de 82,85 %. Cette situation pouvant être à la base des dysfonctionnements rencontrés dans les centres de santé mettant en œuvre la gratuité des soins. Il s’agit entre autres des ruptures de stock des intrants d’où la nécessité de planifier correctement les programmes et de conduire des études d’évaluation d’impact prenant en compte toutes les parties prenantes Au Maroc, Boukhalfa *et al*. ont décrit une participation financière des femmes enceintes concernant aussi bien les accouchements simples que compliqués, avec en outre des paiements informels non retrouvés dans notre série [3]. Au Bénin, Lange *et al*. ont fait un constat similaire dans le cadre de la gratuité de la césarienne, incluant également des paiements informels au bénéfice du personnel soignant [11]. Au Burkina Faso, Méda *et al*. ont rapporté des paiements additionnels d’un montant inférieur au nôtre [13], écart pouvant s’expliquer par un recours plus large aux médicaments génériques dans les centres de santé et de promotion sociale étudiés, par la multiplicité des sites inclus avec d’importantes disparités régionales, et par la non-prise en compte des coûts de transport dans leur travail, autant de facteurs susceptibles de minorer le coût moyen estimé.

Les coûts directs non médicaux (transport, alimentation, communication) occupent une place importante dans les dépenses additionnelles, comme cela a également été observé au Ghana par Anafi *et al*. [1]. Ces frais, souvent minorés dans l’évaluation du fardeau financier de la gratuité, méritent une attention comparable à celle portée aux coûts médicaux.

Le lien entre paiement additionnel et insatisfaction retrouvé dans notre étude est cohérent avec les travaux de Méda *et al*. dans deux autres districts du Burkina Faso [13], ainsi qu’avec ceux d’Anafi *et al*. au Ghana [1]. Dans notre série, cette insatisfaction tenait moins au montant luimême qu’au fait que les femmes ne s’attendaient pas à devoir payer pour des actes et produits normalement couverts par la gratuité.

Ces difficultés persistantes de mise en œuvre ont conduit le ministère de la Santé à prendre, en mars 2024, une mesure de suspension temporaire de la gratuité dans les structures sanitaires privées non confessionnelles, dans le but déclaré de redéfinir les conditions d’inclusion et le dispositif de suivi du programme. Ceci est une illustration des ajustements en cours pour améliorer l’efficience de cette politique.

Ces constats plaident pour la mise en place d’un mécanisme de financement pérenne garantissant une exemption réelle et effective des femmes les plus pauvres, pour un renforcement de l’approvi-sionnement des formations sanitaires en médicaments, consommables et intrants diagnostiques, ainsi que pour une meilleure implication des bénéficiaires et des soignants dans le suivi et l’ajustement du dispositif de gratuité. Cela passe également par le développement des outils pour permettre la rationalisation des prescriptions afin de réduire les dépenses. Il peut s’agir entre autres de logiciels d’aide à la prescription, la prescription de médicaments génériques et des guides de bon usage.

## Conclusion

Notre étude a permis d’évaluer la part addition-nelle payée par les femmes bénéficiaires de la gratuité des soins dans le DGOMR du CHUSS. Nous avons ainsi montré que malgré la mise en œuvre de cette gratuité dans les formations sanitaires publiques du Burkina Faso, il persiste toujours un coût additionnel. Le paiement additionnel à la charge des patientes et de leurs accompagnateurs s’explique par un dysfonctionnement du dispositif de la gratuité, cette situation pouvant compromettre l’utilisation des services de santé par les populations les plus pauvres malgré la mise en œuvre de la gratuité des soins à l’échelle nationale. Le dispositif de gratuité mis en œuvre peine à cibler les ménages les plus pauvres. Cette étude menée dans un seul hôpital du pays nécessite d’être répétée dans les autres formations sanitaires afin d’avoir des données plus exhaustives et de mettre en œuvre des solutions adéquates pour y remédier comme la planification rigoureuse des ressources nécessaires et le développement des outils pour permettre la rationalisation des prescriptions et la bonne gestion des stocks de médicaments.

## Financement

Aucun

## Contribution des auteurs et autrices

TS: conception de l’étude, revue de la littérature, rédaction du document final et correction.

ZCM : conception de l’étude, correction du manuscrit final.

LMBY : collecte des données, revue de la littérature et traitement des données.

## Déclaration de liens d’intérêt

Aucun lien d’intérêt n’a été déclaré.

1. 656 FCFA = 1 euro

## Annexe 1 Questionnaire semi-structuré : Étude du paiement additionnel dans un contexte de gratuité des soins chez les femmes enceintes et accouchées récentes au CHUSS

**Figure:**
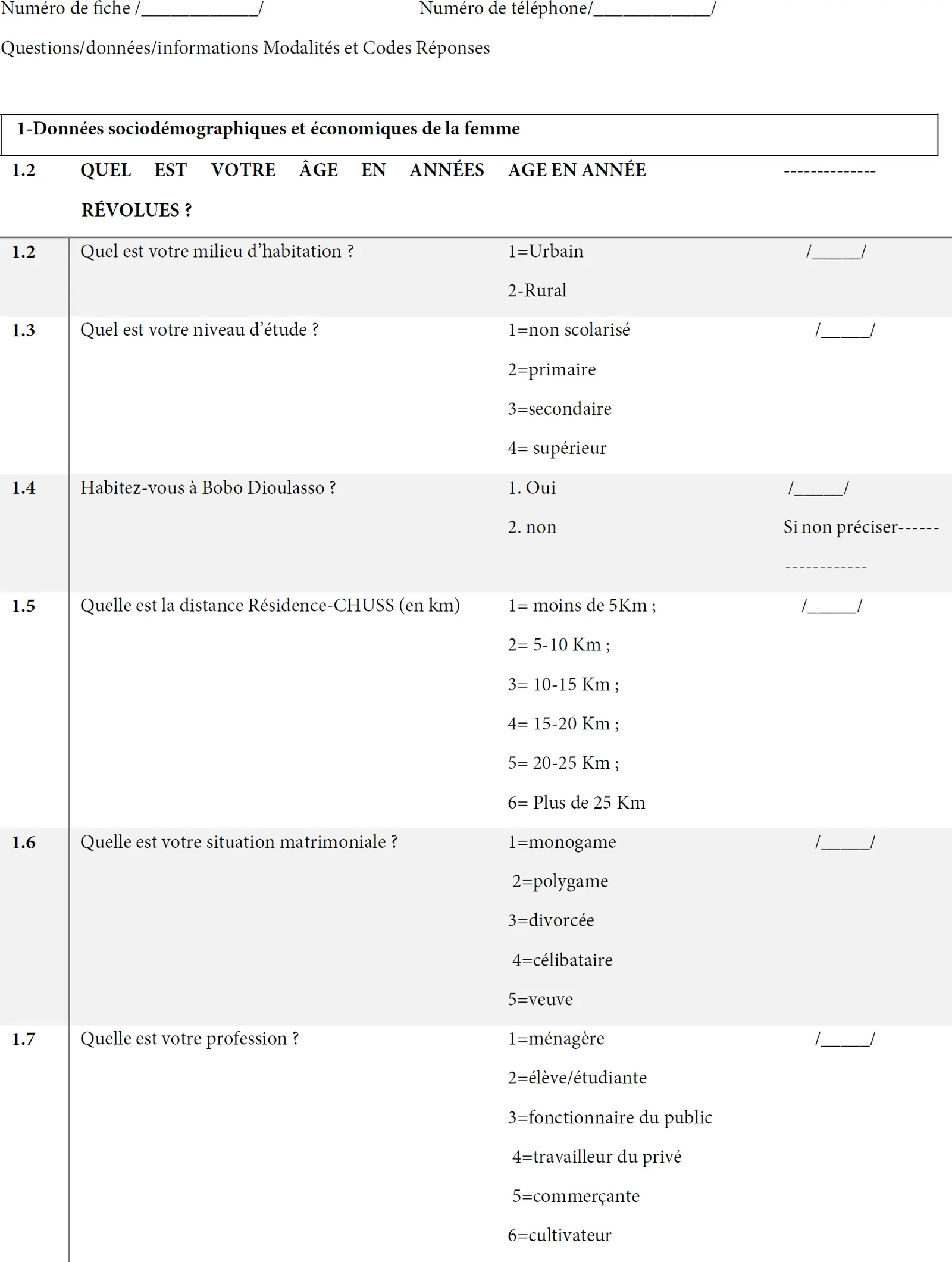

**Figure:**
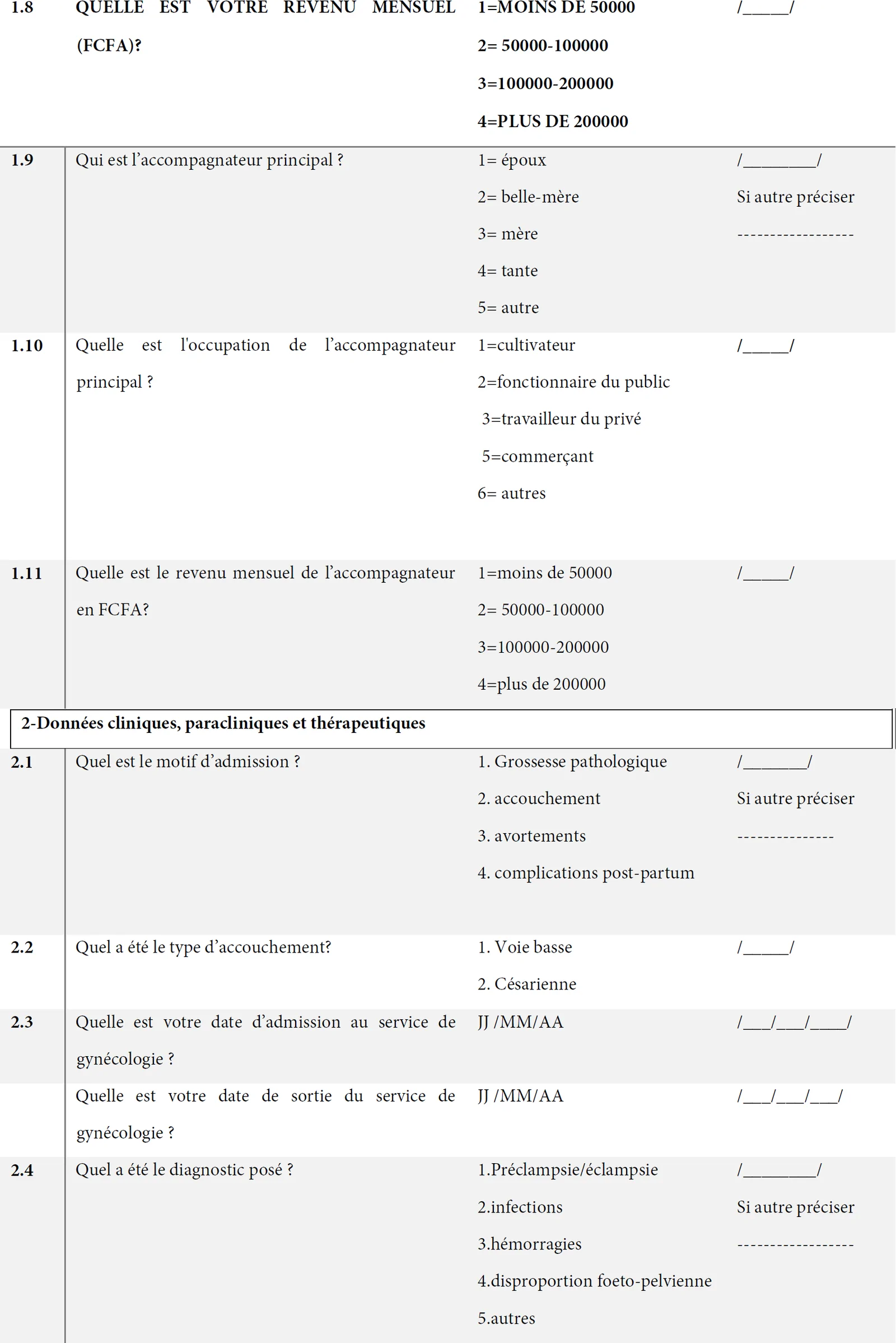

**Figure:**
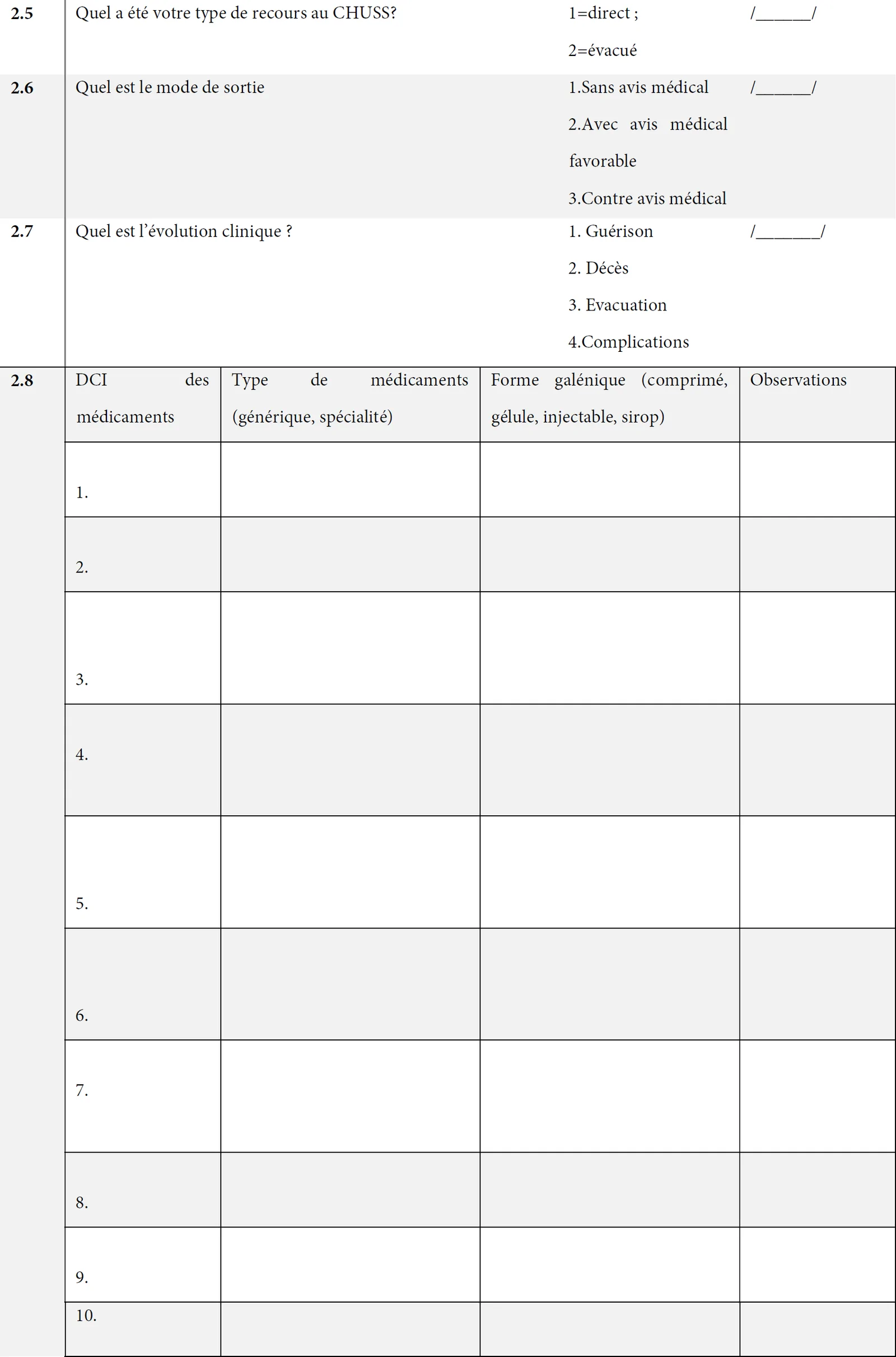

**Figure:**
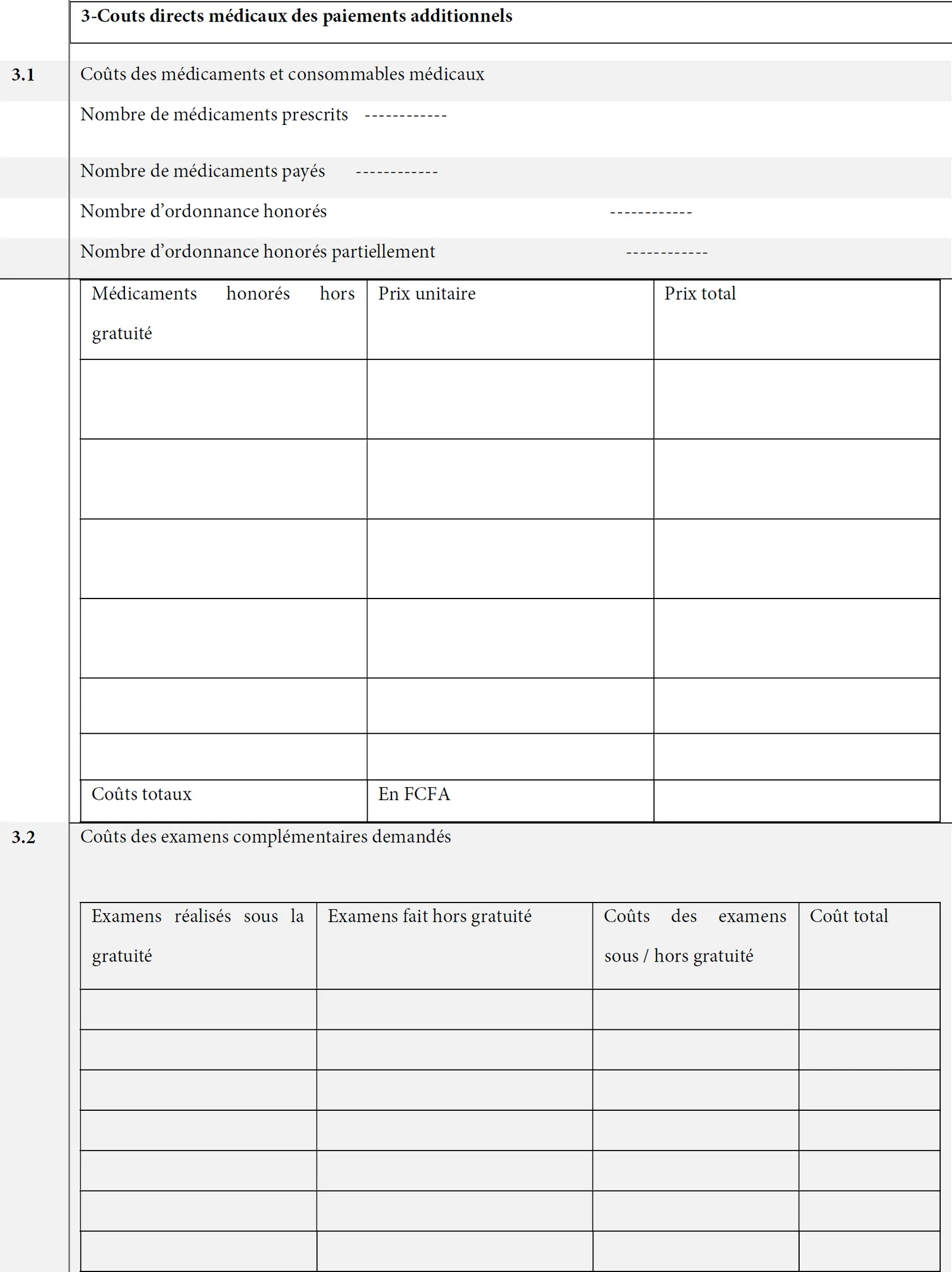

**Figure:**