# Supplementary material for: Additional costs paid by pregnant women receiving free care in the Sourô SANOU University Hospital in Bobo-Dioulasso (Burkina Faso)
Source: Med Trop Sante Int. 2026 Sep 28;6(3):mtsi.v6i3.2026.686. [Article in French] doi: 10.48327/mtsi.v6i3.2026.686 (PMC13697913; doi:10.48327/mtsi.v6i3.2026.686)
Supplement: Related texts

# Research article: Additional costs paid by pregnant women receiving free care in the Sourô SANOU University Hospital in Bobo-Dioulasso (Burkina Faso)

## Introduction

In 1993, Burkina Faso implemented the direct payment system stemming from the Bamako Initiative, which aimed to make districts financially autonomous. Patients were required to pay for consultations and essential medications sold at health center pharmacies. These funds were intended to improve the quality of care by purchasing medications [17].

However, for several years, the country’s health-care system has shown its limitations, with health coverage ranking among the lowest in the world. Among West African countries that adopted the Bamako Initiative, few had more than 0.5 consultations per capita per year [14]. It was imperative to develop initiatives to improve access to care in these countries.

The policy of free healthcare fits within this frame-work. During the 2000s, subsidy and exemption policies proliferated, but they were not often integrated into coordinated programs with long-term visions. Programs providing free healthcare were sometimes implemented merely to secure grants from international institutions [15].

Improving maternal and child health has become a major priority for donors and a full target within the Sustainable Development Goals (SDG 2023) [6].

Burkina Faso has committed to achieving these goals. This requires major health sector reforms. In a country where 45% of the population lives below the poverty line and there is no health insurance system, developing appropriate policies to enable affected populations to access healthcare was necessary [8].

As a result, starting in 2016, the government introduced free healthcare for pregnant women and children under the age of five [14]. This coverage includes medical procedures, hospitalization, tests, and medications. It includes screening for and treatment of precancerous cervical lesions, such as visual inspection after application of acetic acid (VIA) or Lugol’s solution (VIL), cryotherapy, and diathermic loop resection. It also includes the cost of family planning services, including management of side effects related to family planning, as well as care during pregnancy, childbirth, and obstetric procedures. Pregnant women receiving curative care are entitled to all necessary examinations available at health centers offering free services, including transportation costs to the health center [14].

The goal of the free care policy is to increase beneficiaries’ use of health centers [7,16,18]. Despite this policy, some beneficiaries continue to pay fees for care that is normally covered. This occurs when prescribed medications or requested tests are unavailable at the health facility. This forces them to purchase these medications from private pharmacies or undergo the relevant tests at private clinics or laboratories [21].

This study aimed to evaluate the additional costs incurred by pregnant women who receive free care at a specialized Level 3 public facility.

## Materials and methods

This single-center, descriptive study collected prospective data on additional healthcare expenses paid by patients eligible for free care at Sourô Sanou University Hospital (CHUSS) in Burkina Faso. CHUSS is a Level 3 specialized public facility selected for the quality of its curative care within the country’s healthcare system. The objective was to assess these costs from the patients’ perspective. The sample size was determined using the standard formula for estimating a population proportion. In the absence of prior information on the prevalence of interest, the worstcase scenario of 50% was adopted, with a 95% confidence level and a 7% margin of error. This choice was made in the context of an observational study conducted in a hospital setting characterized by a limited source population and logistical constraints on data collection. This resulted in the inclusion of approximately 196 women. Assuming a 10% non-response rate, we estimated the total number of patients to be 220.

Only women who received free medical care on a consecutive basis during the data collection period were included. They were either pregnant or had given birth within the last 42 days, were 18 years of age or older, and were admitted to the obstetrics unit of the Department of Gynecology, Obstetrics, and Reproductive Medicine (DGOMR) at CHUSS. They had to give their consent to participate in the study and complete the data forms. Patients transferred to another department for further care were excluded from the study.

Data were collected prospectively over a one-month period from October 1 to October 31, 2024. Patients were enrolled during their hospital stay. The data collection form was completed daily from admission through discharge regarding daily prescriptions. The total was calculated at discharge.

Figure 1 shows the flow of study participants. Data were collected using a data collection form (see Appendix 1). The questionnaire was administered in paper form and completed during individual interviews with women who were eligible for free care and met our inclusion criteria.

**Figure 1:** Flow of the female beneficiaries interviewed and included in the study The various costs were defined as follows:

Data sources included patient interviews, consultation records, clinical records of hospitalized patients, hospitalization records, payment receipts and invoices, and statements from parents. For healthcare expenses, we reviewed insurance receipts for medications and tests paid for outside the scope of free healthcare.

We calculated average costs by dividing the total costs for a patient by the number of patients surveyed.

Transportation costs were estimated by multiplying the price per kilometer by the number of kilometers traveled by the patient.

- Total direct cost = direct medical costs + direct non-medical costs
- Direct medical cost = cost of diagnosis + cost of treatment
- Cost of diagnosis = cost of diagnostic consultations + cost of diagnostic tests
- Cost of treatment = cost of medications and medical devices used in treatment
- Non-medical direct costs include the cost of transportation to obtain medications and supplies that are normally covered by the free healthcare program but unavailable at the hospital, as well as the costs of food, lodging, phone credit (to contact pharmacies and private laboratories in other cities regarding the availability of covered medications and tests), and other care-related expenses.
- Additional healthcare costs are the total direct costs that should be covered by the free healthcare program, but which are instead borne by patients for various reasons. These costs include direct medical and non-medical costs.

The analysis was conducted using R Studio software and Excel 2019.

Before undertaking this study, we obtained approval from the thesis committee at Nazi Boni University and authorization no. 2024-431/MSHP/ SG/CHUSS/DRH from the CHUSS administration. Participation in the study was freely consented to and verbally confirmed by each patient. Participation did not entitle patients to any benefits, and refusal to participate did not impact the continuity of care.

## Results

The study sample consisted of 220 women. Of those women, 180 had to pay additional costs. For 34 of the women, the additional costs were solely non-medical, while for the remaining 146 women, the additional costs were both medical and non-medical. The mean age was 27 years (95% CI: 25–31), ranging from 18 to 43 years. Eighty-four percent of the women studied lived in urban areas. Those with at most a primary school education accounted for 80.6% of the sample. Most of the women (51.7%) were homemakers, most of whom (82.8%) were married. The primary reason for hospitalization was childbirth (67%). The most common diagnoses were uncomplicated deliveries (38.3%) and cephalopelvic disproportion (28.3%). Table I shows the distribution of patients by primary diagnosis.

Women who presented on their own initiative accounted for 91.1% of the sample. The average hospital stay was 2.9 days (95% CI = 2–3.6), ranging from one to 13 days. Most women were discharged with a favorable medical prognosis (97.8%).

**Table I:** Breakdown of diagnoses made

Diagnoses	Number (n)	%
Fetal-pelvic disproportion	51	28,3
Eutocic delivery	69	38,3
Hemorrhage	3	1,7
Infections	35	19,4
Preeclampsia/eclampsia	10	5,6
Other*	12	6,7
**Total**	**180**	**100,0**

* malaria, dengue fever, typhoid fever

** anemia, spontaneous abortion, sickle cell crisis, diabetes, ectopic pregnancy, molar pregnancy, hypotension, and venous thrombosis

A total of 1,057 prescriptions were issued, 79.6% of which were filled in full and 20.4% of which were filled partially by the patients. A total of 1,874 medications, kits, or medical supplies were prescribed to the 180 patients, with an average of 11.4 items per person. Of the 896 prescribed medications, more than three-quarters were generic drugs (76.1%).

On average, patients paid for 2.2 medications out of pocket (95% CI: 1.64–2.84), ranging from a minimum of one to a maximum of 25.

Patients paid for 267 medications and medical supplies, representing 14.2% of all prescriptions.

The average additional direct medical cost was 7,693.3 CFA francs1 (95% CI = 6,565–8,821), with a median cost of 5,800 CFA francs (IQR = 3,170–10,100). These additional costs accounted for 12.5% of the total direct costs spent on medications and supplies and 2.5% of the total diagnostic test costs. No additional costs were incurred for medical procedures. The average cost of diagnostic tests was 7,477.4 CFA francs (95% CI = 6,359–8,596). Only four tests were performed outside the free program at an average cost of 8,400 CFA francs (95% CI = 4,645–12,154).

Table II shows the breakdown of additional direct medical costs.

**Table II:** Summary of additional direct medical costs in CFA francs

Type of additional direct medical costs	Number (n)	Mean	Standard deviation	95% CI
Total costs of medications and medical supplies covered, excluding those provided free of charge	120	7 477,4	6 250,3	[6 359- 8 595]
Additional total direct medical costs	121	7 693,3	6 331,4	[6 565- 8 821]

Additional costs accounted for 7.2% of total direct medical costs.

Transportation costs for hospitalization, additional tests, and medication purchases accounted for 34% of total non-medical direct costs. The average transportation cost for hospitalization was 1,247 CFA francs (95% CI = 1,125–1,368). Only four patients reported paying for transportation to purchase medications. The average transportation cost for purchasing medications was 879 CFA francs (95% CI = 792–966). Table III details the breakdown of additional non-medical direct costs. The vast majority of patients (94.4%) had their additional costs covered by their companions. Most patients (70%) were not prepared for these costs, and more than half (65.6%) experienced financial difficulties covering them. A large pro-portion of women receiving free care reported that the additional costs were very high (66.7%), and 55.6% expressed dissatisfaction with the services received. The dissatisfied women incurred the highest direct medical and non-medical costs from additional payments. Table IV summarizes this relationship.

**Table III:** Summary of additional direct non-medical costs in CFA francs

Type of additional direct non-medical costs	Number (n)	Mean	Standard deviation	95% CI
Transportation costs for hospitalization in CFA Francs	164	1 246,9	793,9	[1 125-1 368]
Shipping costs for medication purchases in CFA francs	120	879	486,1	[791-966]
Phone charges in CFA francs	84	704	301,8	[639-769]

**Table IV:** Relationship between additional direct medical and non-medical costs in CFA francs and women’s opinions on the services provided

Beneficiaries’ opinions	Number (n)	Average of additional direct medical and non-medical costs	95% CI	p
Unsatisfactory	100	15 418,5	[10 824,1 - 20 012,9]	< 0,001
Satisfactory	80	3 900,4	[2 145,7 - 5 655,1]	
Total	180	10 299,4	[7 682,3 - 12 916,5]	

1. 656 CFA francs = 1 euro

## Discussion

The objective of this study was to estimate the additional costs incurred by pregnant women and received free care at the Sourô SANOU University Hospital in Bobo-Dioulasso.

In Burkina Faso, free healthcare generally covers the screening and treatment of precancerous cervical lesions, family planning, prenatal care, childbirth, obstetric interventions, and necessary diagnostic tests, as well as evacuation costs [14]. In theory, tests performed outside the hospital and medications purchased at private pharmacies by beneficiaries should be fully covered. However, this was not the case in our study.

This discrepancy can be partly explained by the context in which the program was implemented. Decided amid political instability in 2014–2015 and presented as a campaign promise fulfilled, the provision of free healthcare was a political decision, not a technical one. It was implemented without prior consultation with stakeholders, such as healthcare providers, administrators, and beneficiaries. The gradual expansion of the program to private and faith-based healthcare facilities under contract relies on a state reimbursement mechanism that is currently plagued by delays, which jeopardize the restocking of these facilities. The Central Purchasing Agency for Essential Generic Medicines partially mitigates this difficulty by supplying medicines and medical devices on credit. However, the shortages it experiences and the recurring breakdowns of laboratory and imaging equipment regularly force patients to obtain medicines and tests in the private sector. The prospective collection of expenditure data, with verification of supporting documents when-ever possible, is a methodological strength: it has helped limit the recall bias inherent in retrospective surveys of health expenditures.

However, the study has limitations. First, it relies solely on information reported by patients, without systematic cross-checking against the healthcare facilities’ accounting records, which introduces reporting bias. Second, the lack of comprehensive expense records and the variety of caregivers responsible for purchases may have led to an underestimation of direct costs; this bias was mitigated—though not entirely eliminated—by verifying the available supporting documents. Finally, the study was conducted in only one of the country’s five teaching hospitals, which limits the generalizability of the results to the entire country, even though comparable practices have been reported in other facilities. Despite these limitations, we were able to estimate the residual costs paid by beneficiaries of free healthcare.

Our results show that, despite this policy, the vast majority of women had to incur additional expenses—both medical and non-medical—that were significant relative to the resources of the households concerned. This persistence of out-of-pocket payments—which is more frequent and more costly among the least financially secure women—confirms that free care, as currently implemented, does not cover all of the beneficiaries’ needs and, in particular, compromises access to care for the poorest women.

The existence of out-of-pocket payments despite free healthcare policies has been reported in several sub-Saharan African countries, with varying degrees depending on the context. In Ghana, Dalinjong *et al.* linked this phenomenon to a lack of supplies and infrastructure, reflecting insufficient resource allocation to healthcare facilities [5]. In Burkina Faso, the steady increase in expenditures for free health care since its implementation has led to insufficient government budget allocations [20]. In fact, in 2024, the state’s financial coverage rate for free healthcare services was 82.85%. This situation may underlie the operational problems encountered in health centers implementing free healthcare. These include, among other things, shortages of supplies, hence the need to plan programs properly and conduct impact assessment studies that take all stake-holders into account. In Morocco, Boukhalfa e*t al.* described financial contributions by pregnant women for both uncomplicated and complicated deliveries, along with informal payments not observed in our data set [3]. In Benin, Lange *et al.* made a similar observation regarding free cesarean sections, which also included informal payments to healthcare staff [11]. In Burkina Faso, Méda *et al.* reported additional payments of a lower amount than ours [13], a discrepancy that may be explained by the wider use of generic drugs in the health and social welfare centers studied, by the large number of sites included with significant regional disparities, and by the exclusion of transportation costs from their analysis—all factors likely to underestimate the average cost. Direct non-medical costs (transportation, food, communication) account for a significant portion of additional expenses, as was also observed in Ghana by Anafi *et al.* [1]. These costs, which are often underestimated in assessments of the financial burden of free healthcare, deserve attention comparable to that given to medical costs.

The link between out-of-pocket payments and dissatisfaction observed in our study aligns with the findings of Méda *et al.* in two other districts of Burkina Faso [13] and Anafi *et al.* in Ghana [1]. In our study, women were less dissatisfied with the amount they had to pay than with the fact that they had to pay for services and products normally covered by the free healthcare program. Due to these persistent implementation challenges, the Ministry of Health temporarily suspended the free healthcare program in non-denominational private health facilities in March 2024. The stated aim was to redefine the program’s eligibility criteria and monitoring mechanisms. This illustrates the ongoing adjustments being made to improve the efficiency of this policy.

Our findings underscore the need for a sustainable financing mechanism that ensures genuine and effective exemption for impoverished women. This mechanism must also strengthen the supply of medicines, consumables, and diagnostic supplies to health facilities and encourage greater involvement of beneficiaries and healthcare providers in monitoring and adjusting the free healthcare program. It also requires developing tools to streamline prescribing practices to reduce expenditures. These tools may include prescribing assistance software, prescriptions of generic drugs, and guidelines for proper use.

### Conclusion

Our study assessed the additional amount paid by women who are beneficiaries of free healthcare at the CHUSS’s DGOMR. We thus demonstrated that despite the implementation of this free health-care program in Burkina Faso’s public health facilities, an additional cost still persists. The additional payment borne by patients and their companions stems from a breakdown in the free healthcare system; this situation may hinder the use of health services by the poorest populations despite the nationwide implementation of free healthcare. The free healthcare system in place struggles to target the poorest households. The persistence of out-of-pocket costs prevents the mitigation of the financial burden of healthcare expenses. This study, conducted in a single hospital in the country, needs to be replicated in other healthcare facilities to obtain more comprehensive data and implement appropriate solutions to address this issue, such as rigorous planning of necessary resources and the development of tools to rationalize prescriptions and ensure proper management of medication inventories.

### Funding

None

### Authors’ contributions

TS: study design, literature review, drafting of the final document, and proofreading.

ZCM: study design, proofreading of the final manuscript.

LMBY: data collection, literature review, and data analysis.

### Conflicts of interest

The authors declare no conflicts of interest.

1. 656 FCFA = 1 euro

### Appendix: Semi-structured questionnaire: Study of additional payments in the context of free healthcare for pregnant women and women who have recently given birth at the CHUSS
